# Successful percutaneous coronary intervention in a congenital single right coronary artery with acute myocardial infarction: A case report and literature review

**DOI:** 10.1097/MD.0000000000039143

**Published:** 2024-08-02

**Authors:** Bin Li, Jun Lv, Shufang Han, Ruimin Chen, Yuhong Hu, Jie Fang, Zheng Wang, Weiwei Zhong, Yue Hu, Wenyan Liu, Qun Jin

**Affiliations:** a Department of Cardiology, The 960th Hospital of the Joint Service Support Force of the People’s Liberation Army, Jinan, China; b Department of Obstetrics, The Jinan Maternity and Child Care Centers, Jinan, China.; c Department of Emergency, The 960th Hospital of the Joint Service Support Force of the People’s Liberation Army, Jinan, China

**Keywords:** atherosclerotic plaques, computed tomography angiography, coronary angiography, percutaneous coronary intervention, single right coronary artery

## Abstract

**Rationale:**

Single coronary artery (SCA) is a rare coronary artery malformation. SCA combined with atherosclerotic plaques can cause severe and widespread myocardial ischemia and infarction, leading to hemodynamic instability and even sudden death.

**Patient concerns:**

A 48-year-old Chinese man was admitted for treatment of persistent chest tightness and panic for 5 hours. The patient was a lorry driver with high work intensity and mental stress, with body mass index of 33.78, history of smoking and alcohol consumption, but no history of hypertension and diabetes.

**Diagnoses:**

Admission examination showed Troponin was 183.083 µg/L and CK-MB value was >300 µg/L. The patient was diagnosed with a congenital single right coronary artery (RCA) with acute myocardial infarction (AMI) by coronary angiography (CAG). Due to atherosclerotic plaques rupture, a complete occlusion of the proximal RCA with thrombolysis in myocardial infarction grade 0 of distal blood flow were found.

**Interventions and outcomes:**

The patient was treated with thrombus aspiration and thrombolytic therapy by percutaneous coronary intervention under the support of intra-aortic balloon pump. Postoperative the chest tightness and panic were relieved, and CAG revealed that the proximal thrombus of the RCA was reduced, and distal blood flow was restored to thrombolysis in myocardial infarction grade 3. After 2 weeks of intensive antithrombotic and lipid-regulating drug therapy, the patient was successfully discharged. Follow-up for 6 months, the patient was able to live and work normally without experiencing chest tightness and chest pain. Computed tomography angiography (CTA) confirmed a congenital single RCA with patent lumen and no severe stenosis.

**Lessons:**

The congenital single RCA is very rare, and it is fatal in conjunction with acute coronary syndrome. Early detection and appropriate treatment is critical for AMI patient with single RCA. CAG is the gold standard for diagnosis of single RCA, and CTA is a necessary to describe the anatomical course of abnormal coronary arteries.

## 1. Introduction

Single coronary artery (SCA) is a single coronary artery arising from the right, left, or posterior aspect of the sinus of Valsalva through a single coronary ostium and providing perfusion to the entire myocardium.^[1,2]^ SCA is one of the rarest variants of coronary artery anomalies, which carries a potential clinical risk compared to normal coronary artery system.^[3]^ SCA has been known since 1903, the prevalence of SCA in patients undergoing coronary angiography (CAG) has been reported to range from 0.024% to 0.066%.^[4,5]^ Most cases of SCA are asymptomatic, but a proportion of SCA can lead to multiple clinical manifestations, such as angina pectoris, syncope secondary, and cardiac arrest.^[6,7]^ In particular, severe and extensive myocardial ischemia/infarction occurs when SCA combined with atherosclerotic plaques rupture.

To date, the prognosis of SCA is unclear, and there are no guidelines for treating this disease. In general, treatment of patients with SCA includes observation, pharmacological, percutaneous and surgical interventions.^[8,9]^ Revascularization is recommended only in SCA with significant atherosclerosis and documented ischemia. Herein we reported a male patient with congenital single right coronary artery (RCA) who had hemodynamic instability and acute myocardial infarction (AMI) caused by atherosclerotic plaques rupture. The case is extremely rare, we described the diagnostic and therapeutic processes of this case, and provide a brief review of the recent literatures.

## 2. Case report

The patient is a 48-year-old male, who was transferred to Interventional Centre of the 960th Hospital of the Joint Service Support Force of the People’s Liberation Army due to chest tightness and panic for 5 hours. The patient was a lorry driver with high work intensity and mental stress, with body mass index of 33.78, history of smoking (30 packs/yr) and alcohol consumption (100 mL/wk), but no history of hypertension, diabetes, chest tightness and chest pain. The electrocardiogram (ECG) in the outside hospital showed sinus rhythm with high lateral wall ST segment depression in I and aVL leads and ST segment fullness in aVR lead. The initial diagnosis of AMI was made in the outside hospital and the patients was treated with oral Aspirin 300 mg, Tegretol 180 mg and Rosuvastatin 10 mg.

**Figure 1.**
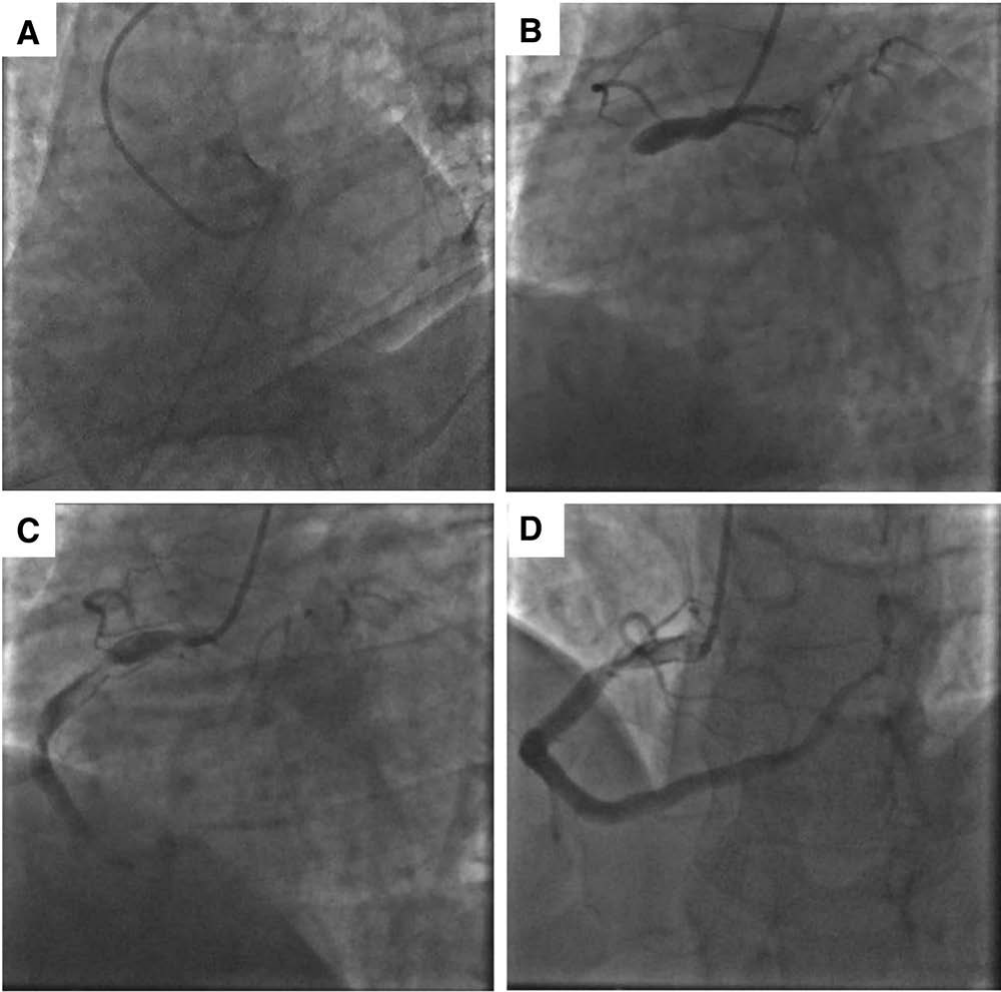
The images of CAG. (A) The left coronary artery was invisible from the left coronary sinus of Valsalva. (B) Abnormally thick RCA with complete occlusion of proximal portion. (C) The RCA was severely stenosed up to 99% with an obvious thrombus shadow by PCI. (D) The proximal thrombus of the RCA was reduced and distal blood flow was restored to TIMI grade 3 after thrombus aspiration and thrombolytic therapy. CAG = coronary angiography, RCA = right coronary artery, PCI = percutaneous coronary intervention, TIMI = thrombolysis in myocardial infarction.

Admission examination showed blood pressure of 85/55 mm Hg, heart rate of 70 beats/min, oxygen saturation of 90% to 95% under nasal cannula oxygenation, and scattered dry and wet rales in both lungs. Troponin was 183 µg/L (reference range: 0–0.034 µg/L) and CK-MB value was >300 µg/L (reference range: 0–5.2 µg/L). Norepinephrine was immediately given to elevate blood pressure by intravenous pumping. Subsequently, the right femoral artery was punctured for emergency CAG, and the left coronary artery was invisible from the left coronary sinus of Valsalva (Fig. 1A), which was considered to be a coronary artery variant. During the operation, the patient grew unconscious and blood pressure declined progressively to 70/40 mm Hg, the contrast catheter was immediately withdrawn. After treated with intra-aortic balloon pump (IABP) through the original right femoral artery access, and the patient was regaining consciousness and blood pressure was increased to 110/70 mm Hg. On the basis of this evidence, the patients was diagnosed with a congenital single RCA with AMI.

The right radial artery was punctured and CAG was performed, and found an abnormally thick RCA with complete occlusion from the proximal end, distal flow thrombolysis in myocardial infarction (TIMI) grade 0, and a right ventricular anterior branch providing partial collateral circulation to the left coronary (Fig. 1B). To treat RCA occlusive lesions, a guidewire was passed through the occluded lesion to reach the distal end and a 2.0 × 10 mm Maverick pre-dilated balloon was used to pass through the occluded lesions, and CAG found RCA abnormally thick with marked proximal thrombus shadow and 99% narrowing of the lumen (Fig. 1C). Thrombus aspiration catheter was then given to aspirate the thrombus and intracoronary injection of recombinant human TNK tissue-type fibrinogen activator 8 mg thrombolytic therapy. Afterward, the patient’s symptom of chest tightness was significantly relieved, and CAG revealed that the proximal thrombus of the RCA was significantly reduced, and distal blood flow was restored to TIMI grade 3 (Fig. 1D).

Postoperative the patient was transferred to the cardiac care unit for hemodynamic monitoring and was administered optimal medical therapy, including dual-antiplatelet therapy (Aspirin: 100 mg/d and Ticagrelor: 90 mg bid), statin (Atorvastatin: 20 mg/d), beta-blocker (Metoprolol: 23.75 mg/d), angiotensin receptor blocker II (losartan: 50 mg/d), and heparin (800–1000 IU/h). After 3 days, the IABP was removed, heparin was replaced by low molecular heparin 0.8 mg subcutaneously. ECG showed sinus rhythm with low and flat T wave in I and avL leads, q wave formation in III and avF leads, ST segment regression in avR lead, and ST segment recovery to baseline level in I and avL leads. Bedside cardiac ultrasound showed left ventricular ejection fraction of 42%, abnormal segmental motion of the left ventricular wall, thickening of the interventricular septum, reduced systolic and diastolic function of the left ventricle, and a large right coronary opening, and the left coronary opening was not detected.

**Figure 2.**
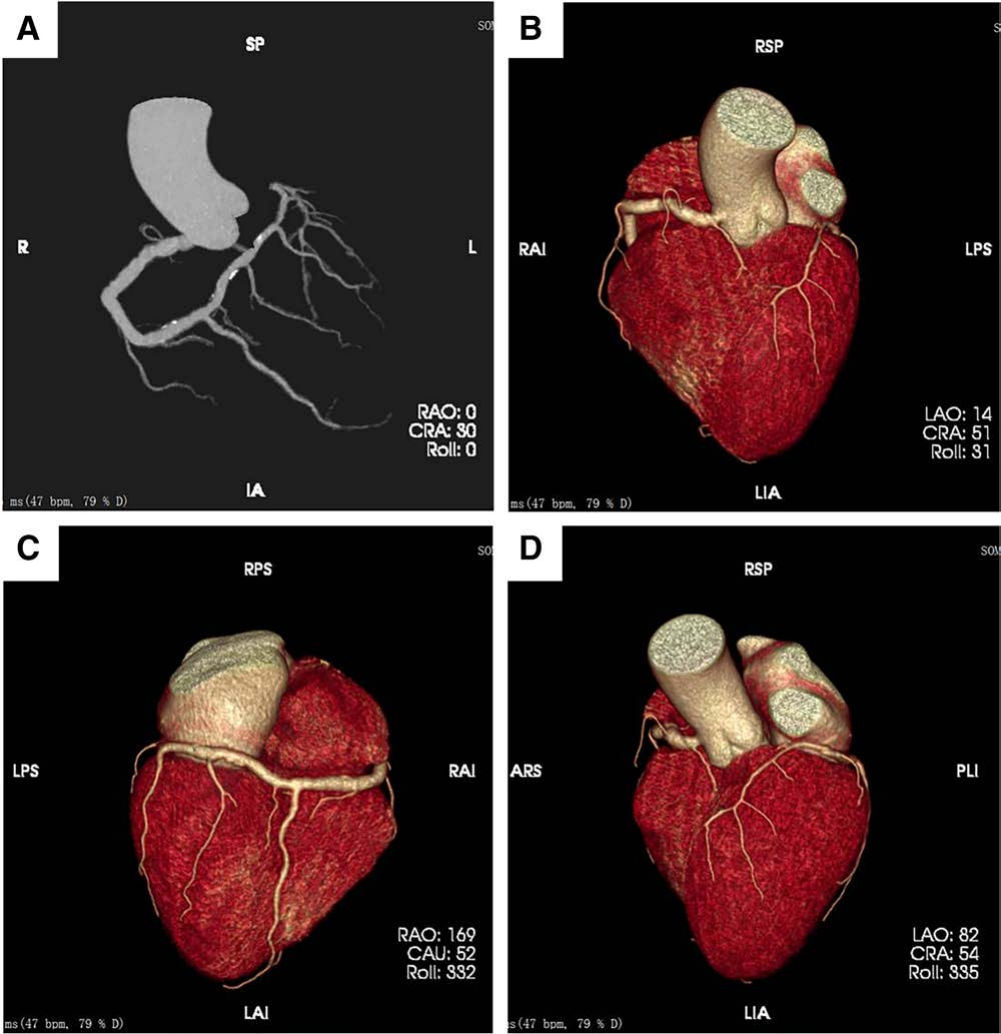
The images of CTA at 6 months after discharge. (A) A single RCA arising from the right coronary sinus with branches supplying the left coronary territory, with patent lumen and no severe stenosis. (B–D) Three-dimensional reconstruction of the anatomical course of the RCA. The RCA emanated from the right sinus and travels in the right atrioventricular groove to the posterior crucifixion where it divided into a posterior descending branch that traveled to the apex and bypasses the apex to supply the posterior interventricular septum and the apical region of the heart, and gave off a diagonal branch that supplied blood to the left ventricular side wall and anterior wall. CTA = computed tomography angiography, RCA = right coronary artery.

The patient was discharged after 2 weeks of intensive antithrombotic and lipid-regulating drug therapy, and continued on antithrombotic medication. Follow-up for 6 months, the patient has recovered well and is able to live and work normally without experiencing symptoms such as chest tightness and chest pain. Computed tomography angiography (CTA) was performed and showed an isolated RCA with scattered calcifications and wall irregularities, but the lumen was patent and there was no stenosis that severely affected blood flow (Fig. 2). A written consent was obtained from the patient for publication of this case report accompanying images.

## 3. Discussion

In 1979, Lipton classified SCA into types I, II, and III based on coronary origin, branching pattern, and disease course.^[10]^ Type I refers to the continuation of the distal segment of a SCA as a larger branch of the contralateral coronary artery, and is divided into 2 subtypes, LI and RI. Type II refers to a SCA emanating from the left or right coronary sinus, there is a larger branch through the root of the aorta to the contralateral side of the normal coronary artery distribution area, according to the branch is located in the right ventricular cone or the pulmonary artery in front of the aorta (A), between the aorta and the pulmonary artery (B), or the root of the aorta after the aorta (P), is divided into subtypes of LIIA, LIIB, LIIP, and RIIA, RIIB, RIIP. The type III originates from the right sinus, with the gyratory and anterior descending branches traveling posteriorly and anteriorly through the aorta, respectively.

In this case, the single RCA was type RI, after the single RCA emanated from the right sinus, the distal right crown sequentially supplied the original anterior descending branch and the area supplied by the gyratory branch. Previous studies have suggested that SCA lacks specific clinical manifestations and can present as dyspnea, chest pain, palpitations or syncope at rest or after activity. ECG of SCA show completely normal, complete left bundle branch block or non-sustained ventricular tachycardia. In this case, the lack of specificity of the ECG presentation may be related to the extensive ischemia caused by the RCA trunk lesion, with the electrocardiographic vector changes canceling each other out.

Current diagnostic methods for SCA included CAG, CTA and coronary magnetic resonance (CMR).^[11]^ Most patients with symptomatic SCA are diagnosed by CAG, which has the advantage of allowing direct management of the coronary lesion, but presents difficulties in identifying the origin and proximal course of the vessels and does not effectively differentiate between coronary artery defects and atresia.^[12]^ CTA has high diagnostic accuracy and is particularly suitable for SCA patients with stable vital signs and atypical symptoms, but has radiation exposure disadvantages.^[13]^ Coronary magnetic resonance has the advantage of not using ionizing radiation and does not require the use of iodinated nephrotoxic ionic or nonionic contrast agents, but has the disadvantage of long acquisition and imaging times.^[14]^ In this case, the patient presented with hemodynamic instability, emergency CAG showed a congenital single RCA with complete occlusion of the proximal RCA with TIMI grade 0 of distal blood flow.

Yamanaka et al classified SAC as a coronary artery anomaly with potential clinical risk.^[4]^ Increasing studies have found that 15% to 45.5% of patients with SCA malformation without atherosclerosis develop angina symptoms, and the patients with SCA without associated atherosclerotic coronary artery disease were managed conservatively with medications and yielded good outcomes.^[15–17]^ Rigatelli et al classified coronary anomalies into 4 categories based on clinical risk, including class A (benign), class B (associated with fixed myocardial ischemia), class C (severe, with the potential to lead to sudden cardiac death), and class D (needing urgent management due to deterioration of the clinical situation).^[18]^ This case belonged to category D, and was treated with thrombus aspiration and thrombolytic therapy under the support of IABP. Postoperative the chest tightness and panic were relieved, and CAG revealed that the proximal thrombus of the RCA was reduced, and distal blood flow was restored to TIMI grade 3. Follow-up for 6 months, the patient was recovering well, and CTA confirmed a congenital single RCA with patent lumen and no severe stenosis.

In conclusion, congenital single RCA is a rare variant of coronary artery anomalies. The single RCA complicated by ruptured coronary atherosclerotic lesions is fatal and requires urgent management. The ECG of single RCA with AMI may lack the dynamic evolution because of the coronary artery anatomical course, and emergent CAG are necessary for early detection. Additionally, single RCA requires echocardiography to rule out other concomitant congenital cardiac anomalies, as well as lifestyle counseling to reduce clinical risk and medication as necessary.

## Author contributions

**Conceptualization:** Bin Li, Jun Lv, Qun Jin.

**Formal analysis:** Shufang Han, Qun Jin.

**Investigation:** Ruimin Chen, Yuhong Hu, Zheng Wang, Weiwei Zhong.

**Methodology:** Jie Fang.

**Resources:** Yue Hu, Wenyan Liu.

**Software:** Bin Li.

**Supervision:** Qun Jin.

**Writing – original draft:** Bin Li.

**Writing – review & editing:** Bin Li, Jun Lv.
